# Pituitary tumour apoplexy within prolactinomas in children: a more aggressive condition?

**DOI:** 10.1007/s11102-018-0900-8

**Published:** 2018-07-16

**Authors:** Elizabeth Culpin, Matthew Crank, Mark Igra, Daniel J. A. Connolly, Paul Dimitri, Showkat Mirza, Saurabh Sinha

**Affiliations:** 1 Departments of Paediatric Neurosurgery, Sheffield Children’s Hospital, Sheffield, S10 2TF UK; 2 Paediatric Neuroradiology, Sheffield Children’s Hospital, Sheffield, S10 2TF UK; 3 Paediatric Endocrinology, Sheffield Children’s Hospital, Sheffield, S10 2TF UK; 4 Paediatric Otorhinolaryngology, Sheffield Children’s Hospital, Sheffield, S10 2TF UK

**Keywords:** Children, Pituitary, Adenoma, Prolactinoma, Apoplexy, Endoscopic, Trans-sphenoidal, Surgery, Haemorrhage

## Abstract

**Objectives:**

To evaluate clinical presentations, diagnosis and management of paediatric patients presenting with pituitary apoplexy.

**Methods:**

A retrospective case series describing a cohort of paediatric patients presenting with this condition from 2010–2016 to a tertiary referral children’s hospital in the United Kingdom.

**Results:**

Pituitary apoplexy is a rare condition that seems to have a higher relative incidence in children than adults. Our series suggests that pituitary apoplexy in paediatric patients with adenomas appears more common than previously described. All our patients required surgery, either as an acute or delayed procedure, for visual compromise. Two patients had commenced growth hormone (GH) for GH deficiency two weeks prior to the onset of pituitary apoplexy.

**Conclusions:**

With only a limited number of published case reports surrounding this topic our case series contributes to help further understand and manage this condition.

## Background

Pituitary adenomas in children are rare with a prevalence of one per million and almost never malignant [1]. They account for < 6% of all adolescent intracranial tumours and < 3% of childhood supra-tentorial tumours [2]. The most common are prolactinomas (50%) occurring most frequently in female adolescents [3].

Pituitary tumour apoplexy is a rare endocrine emergency, resulting from ischaemia and necrosis of a pituitary tumour. There are few case reports [4–6] and no case series detailing apoplexy in children in the current literature, the only data available is from adult studies.

We therefore present a series of paediatric patients presenting with pituitary tumour apoplexy to help better understand this rare condition.

## Case Series

We present a case series of five paediatric patients, (2F, 3M. Age range 13–16 year) all of whom presented as an emergency to a tertiary referral children’s hospital since 2010, with pituitary tumour apoplexy. All the patients were noted to have prolactinomas. Table 1 provides a summary of their presentation.

**Table 1:** Summary of the presentation and management in our case series

Child	Age	Sex	Presentation	Prolactin at presentation miU/L	Age corrected reference range miU/L	Initial management	Timing of surgery
1	16	F	Headaches visual loss amenorrhoea galactorrhoea	10,919	86–324	ETSS	During acute admission
2	14	M	Headaches visual loss	10,626	86–324	ETSS	During acute admission
3	15	M	Headaches visual loss cavernous sinus syndrome	47,173	86–324	Conservative	6 weeks later
4	15	M	Headaches visual loss	67,000	86–324	ETSS	During acute admission
5	13	F	Headaches visual loss amenorrhoea	30,824	102–496	Conservative	16 months later

### Case 1

**Fig. 1:**
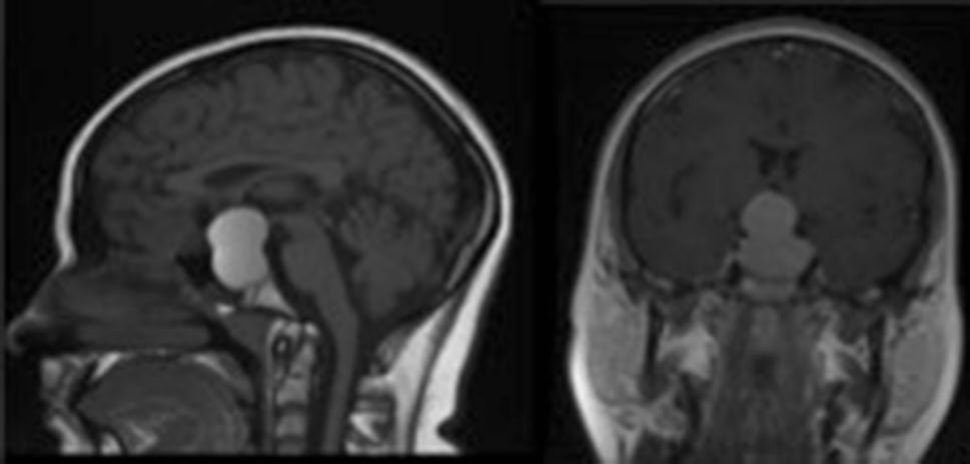
Pre-operative T1 weighted MR image of Child 1 showing a pituitary macroadenoma causing chiasmal compression with extensive high T1 signal within the lesion in keeping with tumour haemorrhage

A 16 year old girl presented with severe headache and visual loss, upon further questioning she also had primary amenorrhoea and galactorrhoea. An MRI [Fig. 1] identified an apoplectic suprasellar tumour with a prolactin of 10,919 miU/L. After a week of treatment with cabergoline her vision had not improved so endoscopic trans-sphenoidal surgery (ETSS) was performed. Post-operatively, her vision returned to normal and she had no further galactorrhoea. 6 years later she has had no recurrence and has a normal prolactin. Histology confirmed pituitary apoplexy with expression of prolactin in a diffuse pattern. The Ki67 proliferation index was low.

### Case 2

A 14 year old boy was admitted with headaches and weight gain. At presentation he had only perception of light in his left eye and hand movements in the right eye. His MRI showed a pituitary tumour extending suprasellarly to compress the chiasm with features of apoplexy. The initial prolactin was 10,626 miU/L. Given his very poor visual function, he underwent emergency ETSS. Post-operatively his vision improved but he remains with significant visual impairment. Histology confirmed a prolactinoma with a moderate Ki67 (5%) and some mitotic activity. He remains on cabergoline although his subsequent MR imaging shows minimal residual disease.

### Case 3

A 15 year old boy was admitted with severe headache, reduced visual acuity and a sixth nerve palsy. Following an MRI he was diagnosed with a large pituitary macroadenoma with features of apoplexy. His prolactin was raised at 47,173 miU/L and he was commenced on cabergoline. His acuity had some improvement, however his sixth nerve palsy remained. Given his on-going visual symptoms, he underwent ETSS 6 weeks later. Post-operatively, his vision returned to normal with no ophthalmoplegia. Histology confirmed a prolactinoma with a low Ki67 and no up-regulation of p53.

### Case 4

A 15 year old boy initially presented with visual deterioration, galactorrhoea and weight gain to another neurosurgical unit. MRI showed a large pituitary tumour. He had a prolactin of 67,000 miU/L and was managed conservatively with cabergoline. His follow-up showed evidence of tumour reduction and prolactin reduced to ~ 10,000 miU/L. He presented to our unit almost a year later with sudden onset severe frontal headache and significant bi-temporal hemianopia. His MRI showed an enlarged apoplectic pituitary tumour with considerable chiasmal compression. Given the severity of his visual deterioration he underwent emergency ETSS. Post operatively his vision improved but recovery was complicated by transient diabetes insipidus, which settled over a few days. Histology confirmed pituitary apoplexy within a prolactinoma. No mitotic activity was seen.

### Case 5

A 13 year old girl presented with a 6 month history of headache and right temporal hemianopia. Her family were also concerned that this previously very studious girl was struggling at school. Her MRI showed a suprasellar apoplectic tumour with a prolactin of 30,824 miU/L. She was started on a dopamine agonist and monitored as an outpatient. During this time her prolactin returned to normal and she was monitored with regular visual field testing and monitoring of her prolactin. After a year she presented again with deteriorating vision. MRI showed an enlarging apoplectic tumour despite a prolactin of 63 miU/L. She therefore underwent ETSS to prevent any further visual loss. Post-operatively, both her vision and her academic performance have returned to normal. Histology confirmed a prolactinoma with apoplexy. The Ki67 and p53 labelling were not elevated.

## Investigations and management

All the children were admitted to our specialist neurosurgical ward within a tertiary children’s hospital and reviewed by a paediatric endocrinologist and a paediatric neurosurgeon. A full endocrine profile was performed as shown in Table 2. All the children were started on hydrocortisone and fluids as per standard apoplexy treatment [7]. Given the raised prolactin levels, cabergoline was commenced in all the children (child 4 was already on cabergoline).

**Table 2:** Showing the pre-operative endocrine profile for our case series

Child	Prolactin miU/L	IGF-1 µg/L	ACTH ng/L	TSH mIU/L	Cortisol mmol/L	FSH iU/L	LH iU/L	Testosterone nmol/L	GH µg/L
1	10,919	264	13.9	0.88	138	2.8	0.1	1.1	0.4
2	10,626	206	25	0.23	23	1.1	1.2	1.6	0.3
3	9939	381	32.2	1.2	111	1.3	2.1	4.8	1.2
4	1368	203	29.8	1.89	190	0.1	0.1	5.8	0.05
5	63	254	12.5	1.71	189	6.0	6.1	0.8	0.1

All the patients had an urgent MRI scan to help evaluate their condition and in all cases this confirmed the diagnosis of pituitary tumour apoplexy.

Figure 1 demonstrates uniform high signal on pre-contrast T1 weighted imaging throughout the large pituitary macroadenoma in keeping with extensive haemorrhage with blood status as methaemoglobin in Child 1.

**Fig. 2:**
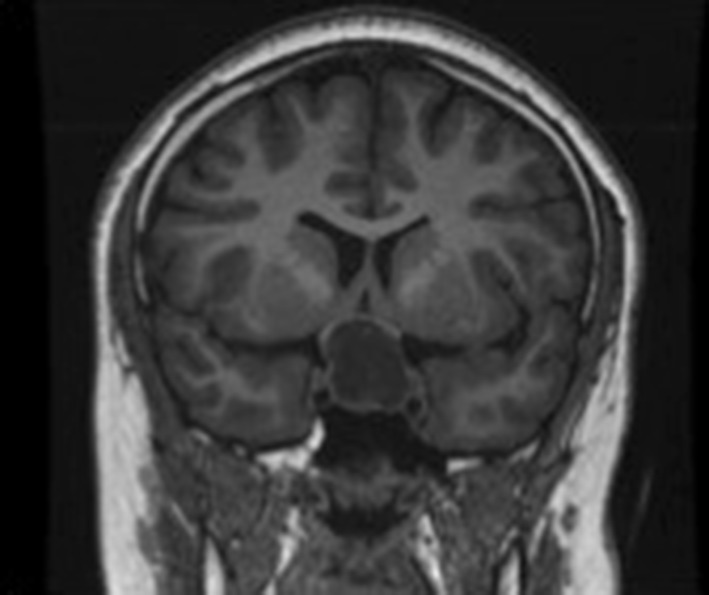
Child 4—Coronal unenhanced T1 MRI showing a tumour splaying the optic chiasm

**Fig. 3:**
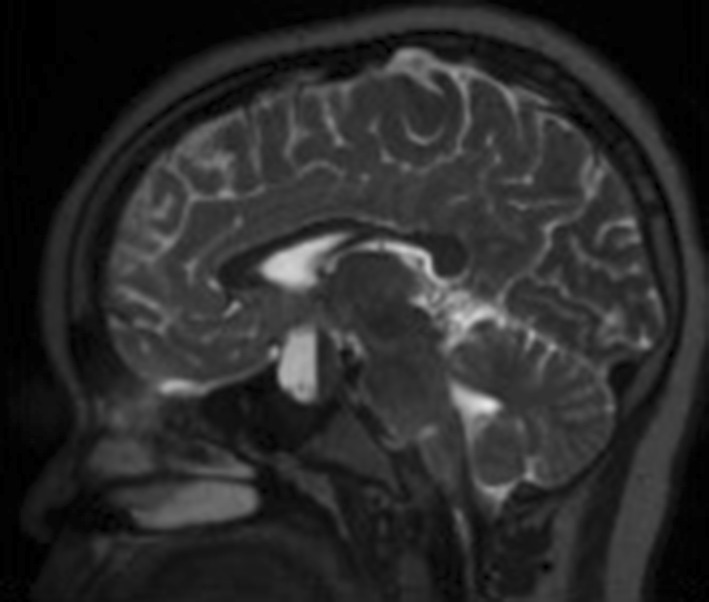
Child 4—Sagittal T2 weighted imaging shows a fluid/blood level in keeping with haemorrhage

**Fig. 4:**
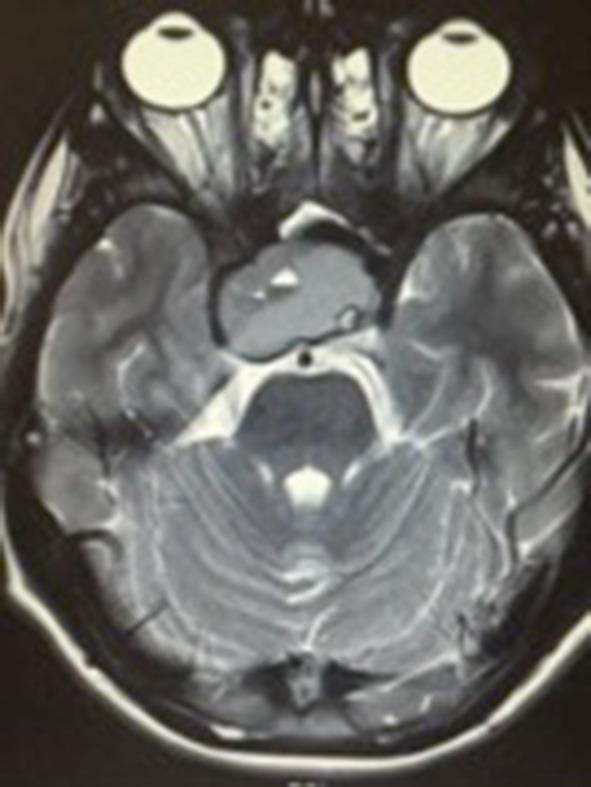
Child 5’s T2 weighted MRI showing a large suprasellar mass with right parasellar extension, displacing the right internal carotid artery. There is evidence of central necrotic change and blood-fluid levels

Blood products of various ages within the mass are shown in child 4 and 5, where there is clear layering in the lesion [Figs. 2, 3, 4].

**Fig. 5:**
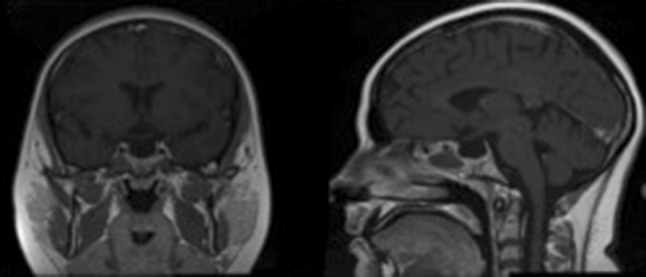
Child 1’s post-operative image showing complete tumour clearance

In all cases the patients had pituitary surgery to preserve visual function. Surgery was performed via an endoscopic trans-sphenoidal approach using the “two nostril, four hand” technique by a paediatric neurosurgeon and otorhinolaryngologist. Following surgery, a MRI scan was performed within 24 h as a baseline—Fig. 5 demonstrates the post-operative imaging in child 1. All the children had post-operative imaging demonstrating a good result from surgery with no on-going chiasmal compression. The patients were monitored in a high dependency unit for 24 h before returning to the specialist neurosurgical ward. After discharge, the children were followed up with a routine combined neuro-endocrine outpatient appointment and open ward attendance if their symptoms were to worsen.

## Discussion

The clinical syndrome of pituitary tumour apoplexy is a medical emergency and requires life saving rapid replacement with hydrocortisone [7]. The decision to manage conservatively or surgically should be made by an experienced multidisciplinary team [8].

In our adult practice, pituitary tumour apoplexy is more commonly seen within non-functioning adenomas. In this paediatric series, however, all the children presented with prolactinomas.

It is now widely recognised that a conservative approach using dopamine agonists (DA) should be used first line in the vast majority of adult prolactinomas presenting with apoplexy, due to a similar efficacy of surgery to conservative management, in terms of visual outcome [9, 10]. In children there are no published guidelines for management but young patients appear to be sensitive to DA. Resistance to DA is associated with higher prolactin levels and larger tumour size [3].

In those who do not respond satisfactorily to conservative management or present with severe visual compromise, surgery may be required. Visual defects should still improve even when delay between onset of symptoms and surgery is long [11]. It has been shown, even in clinically blind patients, that remarkable improvement in vision can be obtained, if surgical decompression of the optic chiasm is undertaken early [12]. Since the initial trans-sphenoidal approach performed in Austria by Schloffer, the trans-sphenoidal approach has become the preferred surgical approach to most pituitary tumours [13].

In our series only one of the five children was already known to have a pituitary macroadenoma (Child 4), the other four children all presented initially with an apoplectic event. One child (Child 5) presented twice with apoplexy, with only the second event requiring surgical intervention.

Pituitary tumour apoplexy in the adult population can also be managed conservatively with surgery being used as a second line treatment for visual compromise. During a similar period, 61 adults presented with apoplexy, 37 (61%) acutely requiring admission and 24 as an out-patient. Only 12 (32%) of the patients admitted to our adult specialist centre needed surgical intervention and in all but one case the underlying tumour was a non-functioning adenoma.

Despite dopamine therapy, our entire paediatric cohort required surgery, either as an emergency or delayed procedure for visual compromise. Histologically, all the children had prolactin-secreting tumours. Proliferation markers showed a low Ki67 in all except Child 2 (moderate ~ 5%) and no up-regulation of p53. Despite the benign histological features the clinical course in our series suggests a more aggressive condition in comparison to adults.

There is no published consensus on how frequently the prolactin level should be checked. There is an argument for annual prolactin levels and repeat imaging only if there is a marked increase in prolactin (> 250 µg/L) or clinical signs of tumour expansion [14].

Although elevated prolactin levels can be secondary to the ‘Stalk effect’ where an expanding mass can interrupt dopamine delivery from the hypothalamus to the pituitary, resulting in loss of inhibition of prolactin release [15], levels of greater than 10,000 would still suggest a prolactinoma despite the lack of correlation between tumour size and the prolactin level. This would be explained by the fact that much of the tumour is necrotic or haemorrhagic and therefore unable to produce the level of prolactin that might be expected of its size.

Only two children (40%) had normal pituitary function pre and post operatively (Child 1 and 3), the other patients all showed some degree of anterior pituitary dysfunction. Child 2 was found to have panhypopituitarism pre-operatively and remained on GH, thyroxine, testosterone and hydrocortisone post-op. Child 4 was found to be deficient in GH, thyroxine and testosterone. Child 5 required GH, thyroxine and hydrocortisone on discharge. This need for hormonal replacement is similar to the adult population.

From an imaging perspective, our case series correlates with the literature related to pituitary tumour apoplexy in the adult population. Typically, non-haemorrhagic, non-cystic adenomas are isointense to grey matter on T1 and T2 weighted imaging [16]. As pituitary macroadenomas enlarge, there is an increased likelihood of necrosis or haemorrhage. Cystic, necrotic areas were seen in four of the cases as well defined areas with low signal on T1 weighted images and high signal on T2 weighted images. MRI characteristics of haemorrhage are variable depending on the status of the haemoglobin, age of haemorrhage and whether there has been a single or multiple episodes of haemorrhage. Diffusion weighted imaging (DWI) can be used to detect areas of infarction within non-haemorrhagic pituitary lesions [17], although susceptibility artefact from adjacent bone structures can make interpretation difficult. Enhancement following administration of gadolinium is variable and may show peripheral, global inhomogeneous or homogeneous focal enhancement [18].

We identified no specific imaging characteristics in the paediatric population that differ from that of adults. All five of our cases demonstrated macroadenomas of 2 cm or greater, several of which had the classic ‘snowman’ appearance as the lesion extends cranially. Child 4 also showed extensive right parasellar extension as well as suprasellar extension, with displacement of the right internal carotid artery. All five lesions caused compression of the optic chiasm.

Two of our patients (Child 4 and Child 5) were started on growth hormone 2 weeks prior to the onset of apoplexy. Although this is not enough evidence for causation, with so little known about precipitating factors to apoplexy, it does merit focus for the future. We believe our data represents the first identification of this potential relationship between starting GH and the onset of apoplexy.

## Conclusion

The low incidence of pituitary tumour apoplexy in children mean there is little published literature surrounding this topic.

Our case series suggests that paediatric pituitary tumour apoplexy is more likely to be seen in adolescents with prolactinomas over 2 cm in size.

We believe our case series suggests that paediatric pituitary tumour apoplexy has a more aggressive natural history in comparison to adults and that early surgery may be more appropriate in this cohort of patients.
